# Healthcare, solidarity, and moral community in Myanmar's humanitarian crisis and revolution

**DOI:** 10.1111/disa.12682

**Published:** 2025-03-18

**Authors:** Anne Décobert

**Affiliations:** ^1^ The University of Melbourne Australia

**Keywords:** citizenship, conflict, disaster, health, humanitarianism, Myanmar, revolution

## Abstract

Drawing on in‐depth qualitative research, this article explores how the experiences and practices of health workers responding to humanitarian crises can foster new forms of solidarity, moral community, and state–society relations in situations of conflict, structural violence, and disputed governance. Focusing on partnerships formed between ethnic minority and former government health workers since Myanmar's coup of 2021, it shows how shared experiences of suffering, and relationships of care forged to address it, foster more inclusive and equitable notions of moral community. Community‐level health partnerships are key not just for crisis response, but also for challenging divisive and exclusionary forms of citizenship, service delivery, and governance, and for building more just and emancipatory models of civic identity, social welfare, and state–society relations—health workers being potential agents of revolutionary change. International aid must support community‐led social welfare mechanisms that build solidarity and social justice ‘from below’ in contexts of structural violence and disputed governance.

## 1 INTRODUCTION

‘All of us are human, right?’, Dr Hnin^1^ murmured, her eyes filled with tears. It was a stiflingly hot and smoggy day on the border with Thailand, far from her hometown in the Bamar heartlands of central Myanmar. Like many other public servants, Dr Hnin—who previously worked at a Ministry of Health and Sports (MoHS)‐run hospital—had refused to keep working for a government taken over by the military, joining the civil disobedience movement (CDM) after the coup on 1 February 2021. When the military began to hunt down the ‘CDMers’, she fled to a border area controlled by an ethnic resistance organisation (ERO). There, she was given shelter and protection by, and started working with, medics from ethnic and community‐based health organisations (ECBHOs).

In a context where decades of conflict and structural violence had crystallised divisive and exclusionary models of civic identity, service delivery, and governance, new partnerships between former MoHS and ECBHO health workers were particularly significant. These affiliations brought together actors from different ethnic and sociopolitical backgrounds and from parallel health and governance systems—with MoHS health workers mainly coming from the dominant Bamar majority in central and urban areas, and having worked under Myanmar state systems, and ECBHO medics coming from ethnic minority groups and para‐state systems in contested borderlands. Like Dr Hnin, many felt that these grassroots‐level partnerships could build more inclusive and just social welfare and governance systems ‘from below’.

As a result of her experiences since the coup, Dr Hnin explained, she now understood better the suffering that ethnic minorities had endured under decades of military rule, and why they were calling for decentralised and more equitable social welfare and governance systems. Although there were daily challenges like language and cultural barriers, she felt that new associations between ethnic minority and former government health workers were fostering much‐needed solidarity, as well as new and more inclusive forms of moral community. After all, Dr Hnin emphasised, people from different ethnic groups and sociopolitical backgrounds were all human and were all suffering the consequences of military violence; and, in coming together to respond to this suffering, they were creating new possibilities to ‘build a better future.’

Drawing on in‐depth qualitative research, this article explores how the experiences and practices of health workers responding to humanitarian crises can foster new forms of solidarity, moral community, and state–society relations in situations of conflict, structural violence, and disputed governance. It delves into the everyday dynamics of Myanmar's ‘revolutionary situation’ (Tilly, 1978; Chambers and Cheesman, 2024)—a time of crisis and rupture marked not only by upheaval, violence, and suffering, but also by the emergence of new alliances, aspirations, and possibilities for change. Focusing on partnerships formed between ethnic minority and former government health workers since Myanmar's coup of 2021, it shows how shared experiences of suffering, and relationships of care forged to address it, foster more inclusive and equitable notions of moral community, and how this may contribute to building more just and emancipatory models of social welfare and state–society relations ‘from below’.

Academics and practitioners have highlighted how disasters and crises can lay bare the boundaries of sociopolitical communities, and the rights and duties associated with constructions of civic identity and belonging. Yet, there has been little empirical research on the everyday experiences and practices of those who—far from being passive ‘victims’—are responding to crises impacting their communities, and how these can transform notions of moral community, social welfare, and governance. As revealed in this article, community‐level health partnerships can be essential not only for crisis response, but also for developing more just and emancipatory models of civic identity, social welfare, and state–society relations in settings historically characterised by divisive and exclusionary forms of citizenship, service delivery, and governance—with community‐level health workers being potential agents of revolutionary change. International actors must support community‐level social welfare systems that build solidarity and social justice ‘from below’ in contexts of protracted conflict, structural violence, and disputed governance.

The article begins by outlining conceptual and methodological approaches to investigating evolving notions of moral community, social welfare, and governance in times of crisis and revolution. It then details research findings, showing: how social welfare systems in Myanmar historically (re)produced divisive and exclusionary ideas of civic identity; how health workers from different systems have established new partnerships and solidarity since the 2021 coup; how experiences of and responses to suffering have been crucial to building solidarity and moral community; and how the latter has contributed to processes of revolutionary change by (re)shaping constructions of civic identity, social welfare, and governance ‘from below’. The article concludes by discussing the transformational potential of health partnerships in Myanmar's revolution, and the implications for international aid.

## 2 MATERIALS AND METHODS

This article examines evolving constructions of moral community and links with crisis and revolutionary dynamics. ‘Moral community’ is conceptualised as shaped by informal constructions of civic identity. These are developed by members of a social group in their everyday lives, including through relationships and practices of service delivery, and they imply shared understandings of who does and does not belong, and of the rights and duties associated with group membership (Morris, 1997; Brubaker, 2010; McCarthy, 2023). The study draws from literature on disasters, crises, and revolutions, and on anthropological and interpretive approaches to analysing experiences and practices pertaining to suffering and social welfare.

### 2.1 Conceptual tools and approaches

Constructions of civic identity can crystallise and (re)produce deep‐seated inequalities and injustices. In countries like Myanmar, where notions of citizenship have been tied to hierarchical ‘national races’, those who do not fit the stereotypical model of ‘citizen’ face high risks of exclusion from social welfare and governance systems—with this being the case even in non‐‘crisis’ periods (Baker and Shryock, 2009). At the same time, even when hierarchical notions of citizenship are enshrined in law, questions of who does and does not ‘belong’ are defined only in part by legal and political regimes. As Brubaker (2010, p. 65) argues, ideas of unity and solidarity are administrated ‘by ordinary people in the course of everyday life, using tacit understandings of who belongs and who does not, of us and them’. As identified through a review of existing literature from Myanmar and as further detailed in the analysis below, social welfare systems can be key sites for the construction and contestation of notions of civic identity and belonging, and for building solidarity across historically divided and unequal groups (McCarthy, 2023; Rain and Naing, 2023; Naing, 2024).

Researchers have also underlined how crises can enhance solidarity, and how solidarity—like social capital—helps communities cope (see, for example, Carroll et al., 2005; Letukas, Oloffsson, and Barnshaw, 2009; Hawdon and Ryan, 2011; Johnson and Buhalis, 2022). In contexts like Myanmar, where the military state drives suffering, what Seekins (2009, p. 732) terms ‘horizontal solidarity as a survival strategy’ becomes vital—with previous research having highlighted grassroots *parahita* ^2^ systems of mutual aid as guarantors of social protection in the absence of adequate state services, and as playing an increasingly important role in times of crisis (Griffiths, 2019; Naing, 2024). But solidarity—again, like social capital (Aldrich, 2012)—can be a ‘double‐edged sword’, excluding those not seen as ‘belonging’. This is all the more so in unequal and exclusionary citizenship regimes (Décobert et al., 2024). Yet crises can also transform notions of solidarity in line with more inclusive forms of moral community.

As highlighted by Fassin and Honneth (2022), crises can ‘lift the lid’ on systemic inequalities and injustices, generating new critiques, identities, alliances, and visions for change. Disasters and crises can transform how individuals and institutions construct the ‘social imaginary’ (Castoriadis, 1997). They can legitimise particular sociopolitical actors and institutions, and generate greater popular expectations of authorities and/or other community members; or, inversely, perceived failures in crisis response can incite critique of and resistance to sociopolitical actors, and lead to the emergence of new forms of solidarity and reciprocity (Seekins, 2009; Remes, 2015; Décobert et al., 2024).

Revolutions can be understood as particular types of crises, implying rupture, uncertainty, and instability, along with ‘a radical negation of the old by the new in the name of a certain idea of progress’ (Myriam Revault d'Allonnes, quoted in Mazeau and Moisand, 2013). They can profoundly reconfigure notions of civic identity, notably through the creation of new understandings and alliances between previously divided groups—with revolutions offering opportunities to disrupt ‘unequal structures of imaginative identification’ (Graeber, 2011, p. 21). As analysed below, Myanmar's revolutionary situation has entailed growing solidarity between different groups, which are increasingly united in the shared goal of ending military rule and which (to some extent at least) have developed greater mutual understanding and empathy (Loong, 2021; Medail and Tun, 2024; Ryan, Tran, and Htut, 2024).

While disaster studies and political science afford us some tools to analyse such transformations, anthropological and interpretive approaches help us to comprehend *how* bonds of horizontal solidarity and notions of moral community are fashioned through the everyday experiences and practices of those impacted by and responding to crises—and how these actors can become key agents of revolutionary change. Anthropologists have underlined how violence and suffering can shape meanings given to, and ways of being in, the world (Kleinman, Das, and Lock, 1997; Morris, 1997; Fassin, 2004). Theories of social suffering and analyses of humanitarian systems alike prompt us to question ‘how such suffering is produced in societies and how acknowledgement of pain, as a cultural process, is given or withheld’, and why the suffering of some is deemed worthy of response, while others are forgotten (Kleinman, Das, and Lock, 1997, p. 15; see also Fassin and Rechtman, 2007). As illustrated below, evolving dynamics concerning the acknowledgement of suffering have shaped responses to Myanmar's humanitarian crisis and contributed to new forms of solidarity and moral community.

Furthermore, analyses of social suffering demonstrate how violence and suffering can not only divide actors into ‘friends’ and ‘foes’, but also can create a shared sense of solidarity and moral community (Morris, 1997; Décobert, 2016). Ethnographic approaches have also highlighted how relationships of care in response to suffering and crises can unite actors, including former ‘enemies’—with the routinised practices and shared lexicon of healthcare in particular providing common ground, from which new understandings, alliances, and possibilities for change are developed (Watkins and Swidler, 2012; Décobert, 2021a). More generally, anthropologists working in conflict and crisis settings have shown that notions of service and duty towards others are forged through processes of building bonds of reciprocity during the provision of aid (Ferguson, 2015; McCarthy, 2023).

This study also builds on recent literature from Myanmar, which explores the wider sense of inter‐ethnic solidarity established within a context of crisis and revolution (see, for example, Loong, 2021; Ryan, Tran, and Htut, 2024), and the role of community‐level social welfare systems in building new notions of ‘humanitarian citizenship’ or civic identity that potentially transcend ethno‐nationalist and other divides (see, for example, Naing, 2024). While there has been much discussion about solidarity in Myanmar, questions remain regarding the extent to which shifts since 2021 have led to genuine recognition and confrontation of deep‐seated patterns of structural violence, and the degree to which they can contribute to shared visions of emancipatory political reform and social justice (Medail and Tun, 2024). Additionally, discussions within academic and practitioner circles tend to assume that evolving constructions of solidarity and moral community through new collaborations between different groups and evolving systems of social welfare are the *effects of* rather than *key drivers of* revolution. This article ‘shifts the script’ in this respect, by analysing community‐level health workers and health partnerships as *key drivers* of revolutionary change, showcasing how the everyday experiences and practices of health workers contribute to building more just and emancipatory models of civic identity, social welfare, and state–society relations ‘from below’.

### 2.2 Methodology

This article draws on relatively recent and longer‐term qualitative research, which can be compared to Burawoy's (2009) extended case methodology—in that it was shaped by long‐term engagement with the context and seeks to connect the micro (health workers' everyday experiences and practices) with the macro (sociopolitical systems and changes). Research for this study comprised interviews and small group discussions with: 33 health workers and leaders of health organisations (14 females and 19 males, of very different ages and seniority levels): doctors, nurses, and administrators who worked for the MoHS before leaving their positions after the 2021 coup; and leaders and medics from the ECBHOs in ERO‐controlled areas. Most interviews and discussions were conducted in person during field research in areas along the Myanmar–Thailand border adjacent to Kayin (Karen) State and Kayah (Karenni) State in March–April 2023; I met with health workers operating inside Myanmar and on the Thai side of the border (where there are training and logistics bases for health workers delivering services in Myanmar). A smaller number of interviews were conducted online during and after this period—for security reasons, and/or to speak with contributors located further inside Myanmar or in exile overseas. The interviews and small group discussions were complemented by ethnographic observation of interactions and working relationships between ECBHO and former MoHS health workers.

Ethics approval for this research was obtained from the Office of Research Ethics and Integrity at The University of Melbourne, Australia, and the Community Ethics Advisory Board in Mae Sot, Thailand. Participants were recruited through purposive and snowball sampling. Interviews were conducted in Burmese, Karen, or Karenni, with the aid of translators, or in English, for those individuals with high‐level English‐language skills owing to their prior training and work. Thematic analysis of interview transcripts and observation notes was performed using the NVivo software package, with themes identified inductively from the data. For ethical and security reasons, no real names or identifying information are included in this article.

This study is further informed by, and was made possible through, my work as a researcher and aid practitioner with health and humanitarian agencies in Myanmar since 2009. This has included extended stints of ethnographic research (comprising participant observation and interviews) with health organisations in Myanmar and on the border over several periods between 2009 and 2021. While widely described as a positive aspect of Myanmar's humanitarian crisis and revolution, new partnerships between ECBHO and former MoHS health workers are sensitive for security and political reasons, and those involved must conceal their activities from the Myanmar military and—when in Thailand—from the Thai authorities. Accessing such individuals relied on working through trusted networks, which take many years to develop. Additionally, to analyse the significance of new health partnerships and their impacts on constructions of moral community, it is essential to understand the history of health and social welfare systems in Myanmar. My previous work on parastate health systems, and on health partnerships during Myanmar's turbulent ‘political transition’, was vital to make sense of contemporary changes (Décobert, 2016, 2020, 2021a, 2021b).

## 3 RESULTS AND ANALYSIS

### 3.1 Structural violence, social welfare, and civic identity

Comprising 135 officially recognised ‘national races’ or ‘ethnic nationalities’ (*taingyintha*) and many unrecognised minorities, Myanmar is a highly diverse and unequal country (Ko et al., 2024). Although notions of ‘ethnicity’ have long been recognised by critical scholars as relational and sociopolitical constructs, primordial and antagonistic understandings of ethnic differences were reinforced through Myanmar's history of colonisation, conflict, and structural violence (Gravers, 1999; Cho, 2018; Oo, 2024). Furthermore, the Bamar majority's institutionalised dominance and exclusion of other ethnic groups from constructions of national identity have been key drivers of conflict and systemic inequalities (Medail and Tun, 2024).

The 1974 Constitution of the Union of Burma institutionalised divisions between the central Bamar majority ‘regions’ and the ‘ethnic states’ along the border areas, which were named after the main ‘ethnic nationality’ group in those states—divisions that crystallised categorisations established under British colonial rule (1824–1948). While the Burmese independence movement had promised autonomy to some of the larger ‘ethnic nationalities’, the period following decolonisation in 1948 saw these groups' hopes for self‐determination dashed. Over the following years, conflict spread in the border areas, as various ethnic armed organisations (EAOs) or EROs (as the former later became known) took up arms against central government control and the state's policy of ‘Bamarisation’. Decades of conflict ensued, with the military state's counterinsurgency policies from the 1960s onwards in particular involving widespread and systematic human rights abuses against civilian populations.

Throughout this time, and even during Myanmar's relative liberalisation in the 2010s, minority groups were systematically marginalised through unequal and exclusionary citizenship, social welfare, and governance regimes (McCarthy, 2023; Ko et al., 2024; Medail and Tun, 2024). While some EAOs/EROs agreed to ceasefires in the 1990s–2000s, political and peace discussions failed to realise ethnic nationality groups' aspirations for self‐determination through federal reform. Unofficial minorities (not recognised as *taingyintha*) also remained excluded from debates about politics, federalism, and power sharing—with these prioritising the relationship between the Bamar majority and officially recognised/territory‐based *taingyintha* (Ko et al., 2024).

Since 1962, Myanmar's diverse populations have also endured decades of oppressive military rule, economic mismanagement, and impoverishment. State disinvestments in health led to the fragmentation, underfunding, and understaffing of public health systems in central, government‐controlled areas (Décobert, Richards, and Thura, 2024), as well as to social welfare and protection being outsourced to private, charitable, and religious groups (Griffiths, 2019; McCarthy, 2023). Meanwhile, populations in border areas lacked access to already overstretched government health systems. And parallel health systems developed under the parastate systems of the EAOs/EROs in areas under their control. Through partnerships between ethnic health organisations (the health departments of the EAOs/EROs) and community‐based organisations, a strong community‐level public health system was established to serve populations in border areas. International donors channelled funding to the resulting ECBHO system to support essential aid for conflict‐affected communities (Décobert, 2016).

Health and welfare systems in different areas were also linked with and (re)produced divisive and exclusionary constructions of civic identity and moral community. Myanmar's Constitution of 2008 embeds ‘national races’ as the basis for full citizenship, fragmenting imaginings of the ‘nation’ through legally enshrined racial hierarchies and lending ‘pseudo‐legal legitimacy to the violent expulsion of minorities, particularly the Rohingya, labelled as “nonindigenous”’ (McCarthy, 2023, pp. 115–116). In Bamar nationalist discourse, Bamar Buddhist groups were pitted against ‘ethnic nationalities’ in border areas, the members of which—as many ECBHO members recounted—were framed as violent and primitive ‘rebels’ and ‘threats to national unity’. In parallel with legal and sociopolitical constructions of civic identity, outsourced social welfare systems in central, government‐controlled areas relied on notions of service and care for others that reproduced narrow, ethno‐nationalist definitions of moral community, marginalising ethnic minorities and excluding groups like the Rohingya—the ultimate ‘other’ (McCarthy, 2020, 2023).

In border areas, notions of civic identity and moral community were also historically linked with social welfare systems that were a product of and a response to a history of structural violence and conflict. Although from diverse geographic areas and ethnic groups, ECBHO medics were brought together by embodied histories of state violence, resulting in a shared ‘black and white’ view of the world: the Bamar‐dominated military state was the source of violence and suffering; and the EROs and their service delivery systems were legitimate governance systems and providers of social welfare (Décobert, 2016). These embodied histories of violence resulted in the conflation of the military state, its army, and the majority Bamar population. A Bamar medical doctor—who, through his work with the MoHS before the coup, had travelled to border areas and met ethnic minority groups—recalled:

*I am Burmese*.^3^ *So, in the ethnic region, Karen region, and people in the ERO‐controlled region, sometimes they are confused between Burma Army and Burmese. They think like that: so, you are Burmese, you are the invader and the killer. So, like Burma Army and Burmese, you are the same*.^4^

Among ECBHO members, a pervasive notion of ‘duty to serve the community’ was also associated with deeply‐embedded perceptions of the Bamar‐dominated military state as not only inefficient, but also abusive and illegitimate. And although institutionally committed to the impartial delivery of care to all, ECBHO members commonly saw their ‘duty to serve’ as centred on narrow, ethno‐nationalist definitions of ‘community’ (Décobert, 2016).

Myanmar's history of structural violence and conflict therefore resulted in parallel health systems, and a lack of trust and collaboration between MoHS and ECBHO health workers. The ECBHOs were not recognised by the Myanmar state—and not only did MoHS workers see ECBHO medics as unqualified, but they also tended to lump them into the categorisation of ‘ethnic rebels’. Conversely, ECBHO medics tended to see MoHS staff as part of ‘the enemy’. As a result of this history, one former MoHS doctor underlined, ‘we have so many barriers—these barriers were not built in one day, but for many, many years’.^5^ Divisions and a lack of trust persisted into Myanmar's contested and uneven ‘political transition’ in the 2010s. During this time, progress in recognition of parastate health systems was limited by a lack of a common vision for the future of Myanmar's social welfare and governance systems, by a lack of trust between those who were part of these systems, and by deeply‐embedded structural violence and centralised governance systems (Décobert et al., 2024).

Yet, even before 2021, health workers and systems—and social welfare systems more generally—held significant potential for dissent. In more central areas, *parahita* groups—that is, social organisations working ‘for the benefit of others’ (Griffiths, 2019)—and private providers of health and other services were key to ensuring social welfare while sustaining critiques of the state (McCarthy, 2023). In border areas, healthcare provision was inextricably linked with what medics described as their struggle for the rights and freedoms of ethnic groups and for federal democracy (Décobert, 2016). Additionally, even before 2021, social welfare systems were potential sites for fashioning more inclusive and equitable notions of civic identity. Community‐level service delivery systems formed by volunteer health workers during the COVID‐19 (coronavirus disease 2019) pandemic contributed to ideas of ‘volunteer citizens’ potentially transcending divisive identity politics; and their networks of solidarity became foundations for popular resistance after the coup, with local systems of social welfare in the absence of a responsive state generating new forms of ‘humanitarian citizenship’ that could contribute to building a more inclusive political community (Rain and Naing, 2023; Naing, 2024). Health was therefore interconnected with the dynamics of dissent, even before Myanmar's current crisis and revolutionary situation. And since 2021, health systems and workers have been key to forging new, revolutionary ideas of moral community, social welfare, and governance.

### 3.2 Health partnerships in crisis and revolution

When General Min Aung Hlaing and his henchman seized power on 1 February 2021, Myanmar was plunged into a multifaceted political and humanitarian crisis. The label of ‘crisis’, however, can be misleading, implying total novelty and ignoring historical dynamics (Fassin and Honneth, 2022). Instead, ‘deeply rooted systems of structural and symbolic violence mean that for many people [in Myanmar], different forms of crisis have always been an everyday state of reality’ (Chambers, 2023, pp. 9–10). Combined with the impacts of COVID‐19, however, the 2021 coup triggered what can be described as a situation of *acute* and country‐wide crisis, characterised by widespread violence and displacement, economic collapse, the breakdown of social services, and exploding humanitarian needs. This is intermixed with a new ‘revolutionary situation’, with ‘*coalitions of contenders*’ advancing ‘exclusive and alternative claims to the current state authority, which *have broad popular support* and that current rulers are *unable to suppress*’ (Medail and Tun, 2024, p. 916; see also Tilly, 1978).

In the days and weeks following the coup, public servants were at the forefront of Myanmar's CDM. Thousands of MoHS staff stopped going to work and took to the streets in peaceful protest. Former MoHS staff explained that, after a decade of relative political liberalisation and greater freedoms, they refused to return to life under military oppression—their dedication during the COVID‐19 pandemic in the face of inadequate state responses having further fuelled their anger at having their votes denied (see also Rain and Naing, 2023). In response to the growing CDM, Myanmar's military occupied medical facilities and hunted down, detained, and killed health workers—Myanmar becoming one of the most dangerous countries in the world in which to be a health worker (Physicians for Human Rights, 2022). Yet, former MoHS staff continued to show amazing courage in spearheading the revolution while continuing to serve local communities. With the official health system crippled and most government facilities barely functional, former MoHS workers continued to provide free services through private clinics, new ‘underground’ facilities, and partnerships with the ECBHOs.

Like Dr Hnin, many former MoHS workers fled for their lives and took refuge in ERO‐controlled areas. There, ERO and ECBHO members supplied them with food, shelter, and protection. Many then started working with ECBHO medics, delivering primary and some secondary health services and training community‐level health workers. These new partnerships took different forms, depending on local needs and established health and governance systems in different areas. Indeed, while former MoHS workers often commented on expecting to find medical wastelands inhabited by fearsome ‘rebels’, what they discovered instead were strong community‐level healthcare systems developed over decades by skilled medics adept at navigating resource shortages and other challenges associated with an unstable context. This meant that, far from entering a void, former MoHS workers had to recognise and fit into or around ECBHO systems.

Of course, this was not the first time in Myanmar's turbulent history that health workers from government‐ and ERO‐controlled areas have come together. After military crackdowns on the 1988 pro‐democracy protests, numerous Bamar medical students and professionals fled to border areas, where they were protected by and formed working relationships with local medics. Partnerships were also built during Myanmar's ‘political transition’ in the 2010s and in response to the COVID‐19 pandemic (Décobert, Richards, and Thura, 2024). Yet, ECBHO members described partnerships since 2021 as unique—not only because of the sheer number of actors from MoHS and ECBHO systems involved, but also owing to the participation of many older and senior professionals and public servants, alongside younger and more junior health workers. This is also significant in that previous analyses of solidarity‐building have highlighted distinctions between a more conservative generation of Bamar leaders and youth‐led progressive groups (see, for example, Medail and Tun, 2024).

New health partnerships, although involving challenges, had numerous practical benefits in terms of improving health systems and outcomes for local communities. They also had significant ‘non‐tangible’ impacts: building solidarity and a new sense of moral community ‘from below’.

### 3.3 Experiences of and responses to suffering

For ECBHO and former MoHS health workers, new partnerships created since 2021 had truly revolutionary potential. The acute crisis precipitated by the coup was a time of opportunity, leading to increased awareness of, and greater will to reform, structures of oppression and inequality. And within this crisis, common experiences of suffering, and new relationships of reciprocity and practices of care forged to respond to this, generated a new sense of moral community and shared purpose between actors historically divided by structural violence and conflict.

Many health workers from central, Bamar‐dominated regions described how, through their experiences in border areas since 2021, they became more aware of the oppression and injustices historically faced by minority communities. Reflecting on the treatment of ethnic minorities by the military state, one Bamar doctor stated that he ‘realised it's very cruel’.^6^ ECBHO members described feeling that their past and ongoing suffering, and the reality of life and work in the border areas, was better understood by Bamar health workers since the coup. Greater understanding of the ‘ethnic other’ also extended to the political goals of ethno‐nationalist groups. A former MoHS worker explained how, in his opinion, the revolution had ‘changed everything’, in terms of how Bamar people viewed ethnic minorities and their calls for federalism:

*I think this revolution changed everything, changed the mindset… Like for example, the Rohingya—before and after, totally changed. We understand that what we knew before was not correct and right… And also, we were not that aware about federalism before. Even when we were young, if you say federalism, this is the word that's going to destroy the country—that's what we understood… But after this coup, I think we matured suddenly.* ^7^

Many ECBHO medics also described becoming increasingly cognisant of difficulties that Bamar people had experienced even before the coup, as well as realising that they could not all be conflated with the military—the source of suffering. In this sense, the crisis precipitated by the coup, and the suffering that it entailed, revealed to diverse actors the structures of oppression within which they all operated, despite ethnic and other differences. Furthermore, it generated opportunities to reinterpret the past and shape new visions of the future (Fassin and Honneth, 2022).

Key to building solidarity was the perception of *shared* experiences of violence and suffering, with this also demonstrating how suffering can forge a sense of solidarity and moral community (Morris, 1997). Putting it bluntly, one ECBHO leader stated: ‘It's not just the ethnic areas now, but the whole of Burma that is struggling’.^8^ A doctor who previously worked in a government‐controlled area reflected how she, along with many of her Bamar friends and colleagues, felt the urge to apologise to ethnic minorities after they also experienced violence and suffering:

*We apologise to the ethnics. Ethnics are our people. We didn't know and we didn't understand their difficulties and their troubles. Because they [Bamar people] felt suffering themselves. Previously they didn't believe the brutal activities of the army. They said the ethnic people said nonsense, and they didn't believe. Now they see, they feel it*.^9^

Although their lives were far from perfect, most Bamar health workers from central and urban areas had little to no direct experience of violence before the coup; and Bamar populations were historically privileged owing to Myanmar's unequal citizenship, social welfare, and governance regimes. Since 2021, however, some of the military's most horrific violence and abuses have taken place in the historically stable Bamar heartlands. And as they faced the military's retaliation against the CDM, many Bamar medical professionals experienced first‐hand the type of suffering and fear that had plagued ethnic minority communities for decades. These experiences not only helped them to understand the historical suffering and political aims of ethnic groups, but they also generated empathy among ECBHO members, who came to see these former government workers as victims of the military, just like themselves. Putting it simply, one ECBHO leader remarked: ‘So, they find difficulty, they ran, they joined the revolution, so they are part of us’.^10^

New relationships of reciprocity and everyday practices of care were also essential to building solidarity. Many Bamar medical professionals remarked on how their views of those they previously saw as fearsome ‘rebels’ had changed, after they were given shelter and protection by ethnic groups. One former MoHS doctor noted that Karen people were ‘very supportive to the CDM people … they welcomed [them] and they provided support’.^11^ Conversely, ECBHO medics commented on their appreciation of the dedication that Bamar health workers had shown by committing their time and scarce resources to serving local communities in ethnic areas. One ECBHO leader stated: ‘the CDMers work together and they are now on the same side, the same with ethnic [groups]’.^12^

Demonstrations of care and commitment were therefore crucial. So too were the daily practices of living and working together. Many Bamar health workers spoke about how the companionship and daily routines of their days in ERO‐controlled areas led them to extend ideas of kinship to those previously seen as the ‘other’. One medical doctor described how he had befriended an ERO officer through shared daily routines: ‘We're just like brothers—I stayed at the military compound, I stayed with him, I bathed with him, I played with him, I ate with him’.^13^ Many other health workers similarly recounted how, by living, eating, and working together, former MoHS and ECBHO health workers developed a mutual understanding and a sense of community. Additionally, a shared focus on patient care and routinised practices of healthcare delivery enabled health workers from different systems to create working relationships, despite historical and practical differences. As a former MoHS doctor working in an ECBHO clinic pointed out, ‘all of us are healthcare providers. I think we somehow have empathy, right? So, we only focus on giving care and improving the patient care’.^14^

Shared experiences of suffering, and everyday relationships of reciprocity and practices of care fashioned in response, were therefore key to building understanding, trust, and solidarity. And this emerging solidarity was integral to Myanmar's revolutionary situation, since it formed the basis for reimagining ideas of moral community, social welfare, and governance.

### 3.4 Reimagining moral community, social welfare, and governance

Myanmar's revolutionary situation has been lauded as the best opportunity the country has had since independence in 1948 to forge a new political order, which would ensure the self‐determination, rights, and freedoms of its diverse peoples. There is arguably now greater unity than ever before between the Bamar majority and diverse ethno‐nationalist groups around the common goals of ousting the military and establishing federalism—although there are also still divisions between ethnic, political, and armed groups (Thawnghmung and Noah, 2021; Medail and Tun, 2024). Mechanisms have also been created to progress discussions between the National Unity Government (comprising elected Members of Parliament ousted by the coup), the EROs, and other stakeholders about the future of Myanmar's health systems—with, notably, the National Unity Constitutive Council's Joint Cooperation Committee–Health taking a lead on policy development, the National Health Committee providing a platform for dialogue between diverse groups, and the Federal Health Professional Council working on health worker recognition—among other mechanisms and agendas. Yet, as even this imperfect summary shows, national‐level coordination instruments are multiple and complex; and local actors point to many challenges linked with ongoing fragmentation, competition, and the diverse agendas of political and armed entities. For local actors, this is precisely why grassroots‐level partnerships are so important, since they can foster solidarity and shared visions of reform ‘from the ground up’, as many put it.

Assumptions of social absence of the state meant that, historically, not only did parallel social welfare systems develop in different areas, but there were also few demands on the state to expand its role (McCarthy, 2023). A lack of faith in the state as an agent of social justice and welfare was pervasive, even during Myanmar's ‘political transition’ in the 2010s (Décobert, 2020). Since 2021, however, not only has there been more critique of the military state, but heightened expectations have also emerged of state actors and institutions. They are increasingly seen as having a political and moral duty to ensure social welfare and justice for Myanmar's diverse peoples (Jordt, Than, and Lin, 2021; McCarthy, 2023).

Many former MoHS health workers who came to work in the border areas after the 2021 coup noted their heightened awareness of the failures of Myanmar's centralised governance systems to ensure effective, equitable, and inclusive health services. A former MoHS doctor recounted:

*I felt sorry when I saw the health system here; it is very different from Yangon and Mandalay. To be honest, I just came to know… They should have better hospitals and great doctors and nurses, with full systems in their regions. They have the right to access the proper health system like us, the people from Yangon, Mandalay*.^15^

Increased critical awareness of the inequitable and exclusionary nature of public health systems in Myanmar, combined with a greater sense of solidarity among health workers from different systems, has shaped evolving notions of moral community and led to more appreciation and questioning of systemic injustices. For many, ideas of moral duty and service have been dissociated from the narrow boundaries of ethno‐nationalist identities; and new relationships of solidarity and reciprocity have led to the ‘other’ being redefined as an equally deserving member of the national community. A young Pa‐O medic highlighted these interconnections:

*Nowadays, the Sayars and Sayarmas* ^16^ *[that is, Bamar doctors] have wider knowledge. These days, all the opinions are changed in the healthcare sector. What I mean is that they respect the essential healthcare need of every human being. In my opinion, all the ethnic people are also people of Myanmar and they [Bamar] are also people of Myanmar too. All the people should have the same medical treatment and the same assistance*.^17^

Interlinked with evolving conceptions of moral community and social welfare was increased recognition of parastate health and governance systems as effective and legitimate systems in ethnic minority areas. Many former MoHS staff highlighted how they had come to appreciate the strengths of the ECBHOs. And while they stressed that all ethnic groups in Myanmar should have equal rights to healthcare, they also emphasised that services in border areas should not be delivered through centralised, Bamar‐dominated systems, but instead through locally‐governed systems adapted to the languages, cultures, and human resources in different contexts. A former MoHS doctor reflected: ‘We should promote their own health system with their own people. Not people from [the central areas] come to their areas and practice. That is not the way. We should empower the local people’.^18^

Many research contributors also suggested that new bonds of solidarity and reciprocity have contributed to evolving understandings of the EROs, which in Bamar nationalist discourse were historically reduced to their armed forces and framed as ‘rebels’ threatening national unity (Oo, 2024)—instead of being seen as legitimate governance systems in ethnic minority areas. Along with greater understanding of the governance role of the EROs, they highlighted greater support for the federal governance systems and self‐determination sought by ethno‐nationalist groups. Echoing the sentiments of many others, a former government health worker expressed his new‐found endorsement of political devolution and power sharing:

*After this revolution, I think we must share our power… There's 135 ethnic [groups] in our country, so we don't know their cultures, we don't know their languages, we don't know their behaviours. So, in Kayin region, as an example, the people who live in this region must take control of everything—the economy, the transportation, the health system*.^19^

In the perceptions of former government health workers, federalism and self‐determination were then reframed: no longer a threat to national unity, federalism was essential to ensure the welfare of and justice for Myanmar's diverse peoples. As another former MoHS doctor explained:

*We need federal health—every sector, we need federal. Because it will be the only solution. As our experience and our knowledge, this centralisation is not okay with this modern situation and this population. Because it cannot solve the problems and it itself is a problem*.^20^

It is important here to note that many of the political discussions around federal reform and power sharing in Myanmar since 2021 have reproduced historical dynamics, whereby unofficial minorities have been excluded through prioritisation of the relationship between the Bamar majority and officially‐recognised/territorially‐based ‘ethnic nationalities’ (Ko et al., 2024). Here again, though, local actors believed that health partnerships could play a role, by promoting changes in beliefs, attitudes, and practices that could in turn foster more inclusive approaches to political reform. Reflecting on how new health partnerships had changed his thinking, one medic told me: ‘*All* levels of people deserve freedom and just governance in Myanmar’.^21^ This statement indicated a widely‐felt shift among health workers from different areas and backgrounds, who came to see themselves as increasingly united in a struggle to ensure the rights and freedoms of *all* people in Myanmar, regardless of ethnic and other differences.

Of course, there remain many challenges to achieving political reform in Myanmar. Research contributors highlighted an ongoing lack of clarity and consensus within and between different groups about what federal health and governance systems would look like in practice. Yet, while there has been progress in some areas in developing new federal systems, many health workers emphasised the need to maintain a certain level of abstractness and ambiguity, so that the important work of establishing solidarity and shared moral values could first take place. In other words, rather than starting by defining the future of Myanmar in terms of what federal systems might look like in their specificity, the priority for many on the ground is to generate new and more inclusive forms of moral community and a broad, shared vision for social justice. And while new forms of solidarity developed from the ground up could to some extent be seen as instrumental—forged from the need to work together towards the relatively short‐term goal of ousting the military from power (Medail and Tun, 2024)—this research demonstrates that local‐level health partnerships are at the same time contributing to building a shared vision of civic identity, transcending ethnic identity politics, and of social justice for Myanmar's diverse peoples.

The findings of this study also have significant implications for international aid. Over the past few decades, international aid to Myanmar has often been fraught and contested; and since 2021, donors and aid agencies have grappled again with complex dilemmas over how to channel assistance in ways that support the long‐term goals of positive peace, equitable development, and social justice. During Myanmar's uneven ‘political transition’ in the 2010s, some international aid interventions were criticised for impacting negatively on conflict dynamics and reinforcing systemic inequalities, by bolstering the Bamar and military elite and by undermining the locally‐grown service delivery and governance systems of ethnic minorities (Décobert, 2021a). More generally, there continues to be a tendency in Myanmar, like elsewhere, for international aid to be top‐down and to reinforce the logics and systems of coloniality, instead of supporting local leadership and systems (Barter and Sumlut, 2023; Kamal, 2023). At the same time, since 2021, civil society leaders have called on international actors to adopt a solidaristic approach—one that would avoid legitimising and reinforcing the position of an illegitimate and abusive military regime, and instead would support locally‐led systems of humanitarian resistance, social welfare, and governance (Ohmar, 2021; Slim, 2022).

There are also positive examples in Myanmar's past, whereby politically‐savvy international aid has supported locally‐led endeavours to foster solidarity and peace ‘from below’ through collaborative health projects (Décobert et al., 2022). Additionally, there have been positive cases of international agencies supporting local leadership in aid and social welfare systems (see, for example, Humanitarian Advisory Group, 2020; HARP‐F, 2022). International actors should learn from such models, and from the current endeavours of local actors who, as well as providing essential services to their communities, are driving Myanmar's revolutionary situation towards emancipation from deep‐seated structures of violence and inequality. And instead of imposing their own visions and programmes, they should recognise and support community‐level social welfare systems that contribute to building solidarity and social justice ‘from below’.

## 4 CONCLUSION

Myanmar's crisis and revolutionary situation has been described as germinating possibilities for profound sociopolitical reform (Jordt, Than, and Lin, 2021; Chambers and Cheesman, 2024). People throughout the country have questioned the very basis of the nation‐state, ideas of citizenship, and the roles of state and non‐state actors in social welfare and justice. Yet, while much has been written about the revolutionary potential of the current circumstances, there has been little focus to date on the role of service providers in this time of crisis, or on *how* the types of solidarity and moral community that can contribute to social justice may be fashioned. Questions also remain about the extent to which emerging forms of solidarity could translate into more inclusive ideas of civic identity and shared goals for social justice—with inter‐group solidarity being probed as potentially instrumental and opportunistic, rather than entailing any genuine commitment to a shared vision for social justice; and with there being notable distinctions between an older and more conservative political leadership and emerging, youth‐led progressive groups (Medail and Tun, 2024; Ryan, Tran, and Htut, 2024). Spotlighting these questions is important not just for Myanmar, but also for other contexts impacted by protracted conflict, structural violence, and disputed governance.

Myanmar's presently acute humanitarian crisis has heightened the role of health workers in challenging and reforming historically fragmented, exclusionary, and unjust social welfare and governance systems. New relationships of solidarity and reciprocity forged between health workers from state and parastate systems—and from different age groups and levels of seniority—have significant potential for building new forms of moral community and civic identity ‘from below’. Brought together through shared experiences of and responses to suffering, health workers have not only continued to serve their communities, but they have also been redefining the very notion of ‘community’, with ideas of belonging, service and duty being extended beyond narrow ethno‐nationalist divides. Building on historical and cultural models of *parahita* (social organisation) and of a ‘duty to serve’ their communities, these diverse health workers have, in coming together, generated new forms of collaboration and disrupted ‘unequal structures of imaginative identification’ (Graeber, 2011, p. 21). At the same time, increased support for federalism—itself an outcome of new partnerships and solidarity—has produced new possibilities for emancipation, with self‐determination of ethnic minority peoples increasingly seen as key to achieving inclusive and just social welfare and governance systems.

Together, these shifts have significant transformational potential in a context where deeply‐embedded patterns of structural violence have driven decades of conflict. They show that, while there are indeed instrumental aspects to new forms of solidarity being created at the grassroots level, these are also contributing to building new notions of moral community and civic identity, which have the potential to transcend ethnic identity politics, and a shared vision of social justice. These changes are particularly important in Myanmar, where the need for a redefinition of national identity has been identified as central to social justice and the development of democratic institutions (Medail, 2021). And looking beyond the country, these shifts highlight the important role of service providers and systems of social welfare in developing more inclusive and equitable ideas of civic identity and state–society relations ‘from below’ in contexts of state violence and disputed governance.

This study also emphasises the importance of interpretive approaches, which enable understanding of *how* political and moral values can be shaped through the lived experiences and practices of those affected by and responding to humanitarian crises—perspectives that typically remain underappreciated in the literature on crises and state–society relations. We then see how, in times of crisis, experiences of suffering, and practices of care developed to react to it, cannot just be reduced to simple impacts on passive ‘victims’ and mechanical responses to the situation. Instead, the experiences and practices of those affected by and responding to humanitarian crises are generative of new ideas and identities, new constructions of ‘self’ and ‘other’, and new aspirations and expectations regarding governing authorities and institutions. As such, we see not only the vital role of health workers in Myanmar's humanitarian crisis and revolution, but also how their experiences and practices create new ideals for the nation‐in‐making, and new possibilities for social justice and emancipation.

Attention to the everyday experiences and practices of those affected by and responding to humanitarian crises is therefore essential to enhance academic understanding of crisis response and processes of revolutionary change. In addition, this is vital to guide international aid in contexts impacted by protracted conflict, structural violence, and disputed governance. Indeed, if they are truly committed to supporting emancipatory political reform and positive peace in countries like Myanmar, it is crucial that international actors recognise and support the new, more inclusive, and equitable forms of moral community, social welfare, and governance being forged by those on the ground.

## ETHICS STATEMENT

This paper reports analysis of primary sources. The ethics of data collection and analysis were approved by The University of Melbourne's Office of Research Ethics and Integrity, Australia, and the Community Ethics Advisory Board, Mae Sot, Thailand.

## FUNDING

This research was funded by the Faculty of Arts at The University of Melbourne, Australia.

## Data Availability

The data that support the findings of this study are available on request from the corresponding author. The data are not publicly available due to privacy or ethical restrictions.

## Endnotes

^1^
Pseudonym. Interview, 16 March 2023, Thailand.

^2^
The Pali term *parahita* can be translated as ‘for the benefit of others’ and is used to describe voluntary social organisations (Griffiths, 2018).

^3^
Many people from Myanmar, when speaking English, use the terms ‘Burmese’ and ‘Bamar’ interchangeably, whereas native English speakers tend to distinguish Burmese (people from the country of Burma/Myanmar) from Bamar (the majority ethnic group). Where the term ‘Burmese’ is included in quotes, it refers to the majority ethnic group.

^4^
Interview, 11 March 2023, Thailand.

^5^
Interview, 19 March 2023, Thailand.

^6^
Interview, 17 March 2023, Thailand.

^7^
Interview, 17 March 2023, Australia.

^8^
Interview, 12 March 2023, Thailand.

^9^
Interview, 6 March 2023, Thailand.

^10^
Interview, 22 March 2023, Thailand.

^11^
Interview, 14 March 2023, Thailand.

^12^
Interview, 6 March 2023, Thailand.

^13^
Interview, 15 March 2023, Thailand.

^14^
Interview, 16 March 2023, Thailand.

^15^
Interview, 16 March 2023, Thailand.

^16^
Sayar and Sayarma literally translate as teacher/leader (male/female, respectively). Here, the medic was referring to Bamar medical doctors.

^17^
Interview, 19 March 2023, Thailand.

^18^
Interview, 14 March 2023, Thailand.

^19^
Interview, 15 March 2023, Thailand.

^20^
Interview, 19 March 2023, Thailand.

^21^
Interview, 19 March 2023, Thailand.

## References

[disa12682-bib-0001] Aldrich, D.P. (2012) Building Resilience: Social Capital in Post‐Disaster Recovery. University of Chicago Press, Chicago, IL.

[disa12682-bib-0002] Baker, W. and A. Shryock (2009) ‘Citizenship and crisis’. In Detroit Arab American Study Team (ed.) Citizenship and Crisis: Arab Detroit after 9/11. Russell Sage Foundation, New York City, NY. pp. 3–32.

[disa12682-bib-0003] Barter, D. and G.M. Sumlut (2023) ‘The “conflict paradox”: humanitarian access, localisation, and (dis)empowerment in Myanmar, Somalia, and Somaliland’. Disasters. 47(4). pp. 849–869. 36484543 10.1111/disa.12573

[disa12682-bib-0004] Brubaker, R. (2010) ‘Migration, membership, and the modern nation‐state: internal and external dimensions of the politics of belonging’. Journal of Interdisciplinary History. 41(1). pp. 61–78.

[disa12682-bib-0005] Burawoy, M. (2009) The Extended Case Method: Four Countries, Four Decades, Four Great Transformations, and One Theoretical Tradition. University of California Press, Berkeley, CA.

[disa12682-bib-0006] Carroll, M.J. , P.J. Cohn , D.N. Seesholtz , and L.L. Higgins (2005) ‘Fire as a galvanizing and fragmenting influence on communities: the case of the Rodeo–Chediski fire’. Society & Natural Resources. 18(4). pp. 301–320.

[disa12682-bib-0007] Castoriadis, C. (1997) The Imaginary Institution of Society. MIT Press, Cambridge, MA.

[disa12682-bib-0008] Chambers, J. (2023) ‘Myanmar in crisis’. In J. Chambers and M.R. Dunford (eds.) Myanmar in Crisis: Living with the Pandemic and the Coup. ISEAS Publishing, Singapore. pp. 3–28.

[disa12682-bib-0009] Chambers, J. and N. Cheesman (2024) ‘Introduction: revolution and solidarity in Myanmar’. Journal of Contemporary Asia. 54(5). pp. 741–758.

[disa12682-bib-0010] Cho, V. (2018) ‘Ethnicity and identity’. In A. Simpson , N. Farrelly , and I. Holliday (eds.) The Routledge Handbook of Contemporary Myanmar. Routledge, Abingdon. pp. 43–51.

[disa12682-bib-0011] Décobert, A. (2016) The Politics of Aid to Burma: A Humanitarian Struggle on the Thai‐Burmese Border. Routledge, Abingdon.

[disa12682-bib-0012] Décobert, A. (2020) ‘“The struggle isn't over”: shifting aid paradigms and redefining “development” in eastern Myanmar’. World Development. 127 (March). Article number: 104768. 10.1016/j.worlddev.2019.104768.

[disa12682-bib-0013] Décobert, A. (2021a) ‘Health as a bridge to peace in Myanmar's Kayin State: “working encounters” for community development’. Third World Quarterly. 42(2). pp. 402–420.

[disa12682-bib-0014] Décobert, A. (2021b) ‘Partnerships for universal health coverage in Myanmar: power and politics within “immunisation encounters” in Kayah State and Kayin State’. The Journal of Development Studies. 57(8). pp. 1267–1281.

[disa12682-bib-0015] Décobert, A. , A. Richards , and S. Thura (2024) ‘Public health: state and non‐state systems in a changing society’. In A. Simpson and N. Farrelly (eds.) Myanmar: Politics, Economy and Society. Routledge, Abingdon. pp. 191–206.

[disa12682-bib-0016] Décobert, A. , T. Traill , S. Thura , and A. Richards (2022) ‘How political engineering can make health a bridge to peace: lessons from a Primary Health Care Project in Myanmar's border areas’. *BMJ* Global Health. 7(S8). Article number: e007734. https://gh.bmj.com/content/7/Suppl_8/e007734. 10.1136/bmjgh-2021-007734 PMC9535148 36210071

[disa12682-bib-0017] Décobert, A. et al. (2024) ‘We returned home empty‐handed: COVID‐19, care, and contested citizenship of Naga migrant workers in northeast India’. Disasters. 48(3). Article number: e12616. 10.1111/disa.12616. 38098173

[disa12682-bib-0018] Fassin, D. (2004) ‘Et la souffrance devint sociale. De l'anthropologie médicale à une anthropologie des afflictions’. Critique. 680–681(1–2). pp. 16–29.

[disa12682-bib-0019] Fassin, D. and A. Honneth (2022) Crisis Under Critique: How People Assess, Transform and Respond to Critical Situations. Columbia University Press, New York City, NY.

[disa12682-bib-0020] Fassin, D. and R. Rechtman (2007) L'empire du traumatisme. Enquête sur la condition de victime. Flammarion, Paris.

[disa12682-bib-0021] Ferguson, J. (2015) Give a Man a Fish: Reflections on the New Politics of Distribution. Duke University Press, Durham, NC.

[disa12682-bib-0022] Graeber, D. (2011) Revolutions in Reverse: Essays on Politics, Violence, Arts, and Imagination. Autonomedia, New York City, NY.

[disa12682-bib-0023] Gravers, M. (1999) Nationalism as Political Paranoia in Burma: An Essay on the Historical Practice of Power. Curzon Press, Richmond.

[disa12682-bib-0024] Griffiths, M. (2019) ‘Networks of reciprocity: precarity and community social organisations in rural Myanmar’. Journal of Contemporary Asia. 49(4). pp. 602–625.

[disa12682-bib-0025] HARP‐F (Humanitarian Assistance and Resilience Programme Facility) (2022) Localisation Approach of Humanitarian Protection Responses in Myanmar. June. https://www.harpfacility.com/resources/localisation-approach-humanitarian-protection-resp/ (last accessed on 12 February 2025).

[disa12682-bib-0026] Hawdon, J. and J. Ryan (2011) ‘Social relations that generate and sustain solidarity after a mass tragedy’. Social Forces. 89(4). pp. 1363–1384.

[disa12682-bib-0027] Humanitarian Advisory Group (2020) Localisation through Partnership: Shifting Towards Locally‐Led Programming in Myanmar. Phase 2 – Navigating the Transition. August. Humanitarian Advisory Group, Melbourne, VIC.

[disa12682-bib-0028] Johnson, A‐G . and D. Buhalis (2022) ‘Solidarity during times of crisis through co‐creation’. Annals of Tourism Research. 97 (November). Article number: 103503. 10.1016/j.annals.2022.103503

[disa12682-bib-0029] Jordt, I. , T. Than , and S.Y. Lin (2021) How Generation Z Galvanized a Revolutionary Movement against Myanmar's 2021 Military Coup. Trends in Southeast Asia. 7. ISEAS Publishing, Singapore.

[disa12682-bib-0030] Kamal, A. (2023) The Humanitarian Leader. Beyond the ‘Egosystem’: A Case for Locally Led Humanitarian Resistance. Working Paper 041. November. Centre for Humanitarian Leadership, Deakin University, Burwood, VIC.

[disa12682-bib-0031] Kleinman, A. , V. Das , and M. Lock (1997) ‘Introduction’. In A. Kleiman , V. Das , and M. Lock (eds.) Social Suffering. University of California Press, Berkeley, CA. pp. ix–xxv.

[disa12682-bib-0032] Ko, A.K. , E.L. Rhoads , N. Tinilarwin , W.B. Aung , and Y.T. Khaing (2024) ‘Beyond federalism? Inclusion, citizenship, and minorities without territory in Myanmar's Spring Revolution’. Journal of Contemporary Asia. 54(5). pp. 938–961.

[disa12682-bib-0033] Letukas, L. , A. Oloffsson , and J. Barnshaw (2009) Solidarity Trumps Catastrophe? An Empirical and Theoretical Analysis of Post‐Tsunami Media in Two Western Nations. Preliminary Paper #363. Disaster Research Center, University of Delaware, Newark, DE.

[disa12682-bib-0034] Loong, S. (2021) Centre–Periphery Relations in Myanmar: Leverage and Solidarity after the 1 February Coup. Trends in Southeast Asia. 9. ISEAS Publishing, Singapore.

[disa12682-bib-0035] Mazeau, G. and J. Moisand (2013) ‘Revolution and the crisis of temporality: an interview with Yves Citton and Myriam Revault d'Allonnes’. Books & Ideas website. 27 May. https://laviedesidees.fr/Revolution-and-the-Crisis-of#:~:text=%E2%80%9CYou%20trust%20in%20the%20present,perspective%20on%20the%20historical%20future (last accessed on 12 February 2025).

[disa12682-bib-0036] McCarthy, G. (2020) ‘Bounded duty: disasters, moral citizenship and exclusion in Myanmar’. South East Asia Research. 28(1). pp. 13–34.

[disa12682-bib-0037] McCarthy, G. (2023) Outsourcing the Polity: Non‐State Welfare, Inequality, and Resistance in Myanmar. Cornell University Press, Ithaca, NY.

[disa12682-bib-0038] Medail, C. (2021) Towards an Inclusive Multinational Society: Ethnic Perspectives on State Building. Unpublished PhD (Doctor of Philosophy) thesis. University of New South Wales, Sydney, NSW.

[disa12682-bib-0039] Medail, C. and S.C.T. Tun (2024) ‘Instrumentalism or commitment to social justice? Shifting inter‐ethnic solidarities in post‐coup Myanmar’. Journal of Contemporary Asia. 54(5). pp. 913–937.

[disa12682-bib-0040] Morris, D.B. (1997) ‘About suffering: voice, genre and moral community’. In A. Kleiman , V. Das , and M. Lock (eds.) Social Suffering. University of California Press, Berkeley, CA. pp. 25–46.

[disa12682-bib-0041] Naing, A. (2024) ‘Humanitarian activism, social protection, and emergent citizenship in Myanmar’. IDS Bulletin. 55(2). 10.19088/1968-2024.125.

[disa12682-bib-0042] Ohmar, K. (2021) ‘There's nothing neutral about engaging with Myanmar's military’. The New Humanitarian website. 28 July. https://www.thenewhumanitarian.org/opinion/2021/7/28/theres-nothing-neutral-about-engaging-with-myanmars-military (last accessed on 12 February 2025).

[disa12682-bib-0043] Oo, N. (2024) ‘Shades of identity politics in Myanmar’. The Myanmar Thinkpiece website. 5 August. https://www.mythinkpiece.org/post/shades-of-identity-politics-in-myanmar (last accessed on 12 February 2025).

[disa12682-bib-0044] Physicians for Human Rights (2022) ‘415 attacks on health care in Myanmar during one year of crackdowns following military coup: report’. Website. 26 January. https://phr.org/news/415-attacks-on-health-care-in-myanmar-during-one-year-of-crackdowns-following-military-coup-report/ (last accessed on 12 February 2025).

[disa12682-bib-0045] Rain, S. and A. Naing (2023) ‘Emergent identity: ‘Coupvid’, volunteerism, and the new citizen in Myanmar’. In G. Houtman and Chayan Vaddhanaphuti (eds.) Triple Crisis in Myanmar: Coup, Covid & Climate Change. Regional Center for Social Science and Sustainable Development, Chiang Mai. pp. 39–66.

[disa12682-bib-0046] Remes, J.A.C. (2015) Disaster Citizenship: Survivors, Solidarity, and Power in the Progressive Era. University of Illinois Press, Champaign, IL.

[disa12682-bib-0047] Ryan, M. , M.V. Tran , and S.Y. Htut (2024) ‘Strange bedfellows or trusted comrades? Digital solidarity building among Myanmar's revolutionaries’. Journal of Contemporary Asia. 54(5). pp. 887–912.

[disa12682-bib-0048] Seekins, D. (2009) ‘State, society and natural disaster: Cyclone Nargis in Myanmar (Burma)’. Asian Journal of Social Science. 37(5). pp. 717–737.

[disa12682-bib-0049] Slim, H. (2022) ‘Humanitarian resistance: its ethical and operational importance’. Humanitarian Practice Network website. 20 September. https://odihpn.org/publication/humanitarian-resistance-its-ethical-and-operational-importance/ (last accessed on 12 February 2025).

[disa12682-bib-0050] Thawnghmung, A.M. and K. Noah (2021) ‘Myanmar's military coup and the elevation of the minority agenda?’. Critical Asian Studies. 53(2). pp. 297–309.

[disa12682-bib-0051] Tilly, C. (1978) From Mobilization to Revolution. Random House, New York City, NY.

[disa12682-bib-0052] Watkins, S.C. and A. Swidler (2012) ‘Working misunderstandings: donors, brokers, and villagers in Africa's AIDS industry’. Population and Development Review. 38(S1). pp. 197–218.

